# Covalent Interaction Between High-Amylose Corn Starch and Ferulic Acid: Reshaping of the Structure

**DOI:** 10.3390/foods15122236

**Published:** 2026-06-21

**Authors:** Jiayue Wang, Junqing Zhang, Aoyang Qu, Qingfeng Zhang, Nuo Xu, Biqi Liu, Xinyan Yang, Ning Xu, Ling Guo, Yujun Jiang, Jianguo Sun

**Affiliations:** 1 Key Laboratory of Dairy Science, Ministry of Education, College of Food Science, Northeast Agricultural University, Harbin 150030, China; 15844469223@163.com (J.W.); quaoyang0121@163.com (A.Q.); 15851483990@163.com (Q.Z.); xunuo15545167682@163.com (N.X.); 13206658679@163.com (B.L.); yangxinyan@neau.edu.cn (X.Y.); 2 Heilongjiang Feihe Dairy Co., Ltd., Qiqihar 164800, China; zhangjunqing@feihe.com; 3 Key Laboratory of Infant Formula Food, State Administration for Market Regulation, Harbin 150030, China

**Keywords:** high-amylose corn starch, ferulic acid, covalent grafting, in vitro digestibility, molecular dockin

## Abstract

This study investigated the covalent grafting of ferulic acid (FA) onto high-amylose corn starch (HACS) through controlled moist heat treatment as a strategy to regulate starch structure and digestibility. Fourier transform infrared spectroscopy (FTIR) and proton nuclear magnetic resonance (^1^H NMR) analyses confirmed the formation of ester linkages between HACS and FA. Scanning electron microscopy (SEM) revealed that FA grafting induced a rougher granule surface and increased porosity, while differential scanning calorimetry (DSC) and thermogravimetric analysis (TGA) indicated altered gelatinization behavior and thermal stability. In vitro digestion analysis showed that the rapidly digestible starch content decreased from 23% to 15%, whereas the resistant starch (RS) content increased to 48% after FA grafting. Molecular docking suggested that FA could interact with α-amylase and that covalent modification may reduce enzyme accessibility to starch chains, thereby limiting starch hydrolysis. These findings demonstrate that FA grafting effectively reshapes the structural and digestive properties of HACS and provides a promising approach for developing resistant starch-rich functional food ingredients.

## 1. Introduction

Starch is a primary carbohydrate source in the diet, valued for its versatility, widespread availability, low cost, renewability, and biodegradability [1]. Among the various starch sources, corn starch is the most widely produced, accounting for approximately 80% of global starch production [2]. Conventional corn starch generally contains 20–40% amylose, whereas high-amylose corn starch (HACS) exceeds 50% [3]. HACS is a key feedstock for resistant starch production and is commonly used in developing low glycemic index foods [4]. Relative to conventional corn starch, HACS exhibits higher crystallinity and tighter chain packing, making it less prone to enzymatic hydrolysis and more digestion-resistant [5,6]. Despite these advantages, such intrinsic structural features impose significant constraints on the practical applications of HACS. Specifically, its high gelatinization temperature increases energy demand during thermal processing [7], its ordered, compact crystallinity also hinders diffusion, resulting in poor solubility in cold water and common solvents [8]. Consequently, there is increasing attention to modification approaches that tailor starch properties to better control digestibility while enhancing processing performance.

To date, starches from different sources (e.g., corn, wheat, and pea) have been structurally modified using physical, enzymatic, and chemical approaches, including esterification [9,10,11]. Although these methods can effectively modify the physicochemical properties of starch, chemical approaches are often limited by concerns regarding chemical residues, whereas enzymatic and physical methods may be restricted by substrate accessibility and processing efficiency [12]. Consequently, researchers have gradually shifted toward greener and safer modification approaches, and phenolic acids have attracted growing attention as natural, non-toxic modifiers [13]. Previous studies have shown that gelatinization behavior, rheological properties, and in vitro digestibility can be modulated by interactions between starch and phenolic acids [14,15]. However, many of these systems are based on non-covalent associations, which may be insufficiently stable under rigorous food processing and changing gastrointestinal conditions. Therefore, establishing stable covalent linkages between phenolic acids and high-amylose starch during food processing is important for understanding and regulating starch modification. As summarized in Table 1, previous studies have explored starch modification with phenolic acids or polyphenols through non-covalent complexation, inclusion complex formation, high-pressure homogenization, and esterification. These studies showed that phenolic compounds can slow starch digestion, reduce predicted glycemic index, increase resistant starch content, or enhance antioxidant properties. Nevertheless, most reported systems have focused on non-covalent complexes, starches from different botanical sources, or selected functional properties, whereas covalent modification of high-amylose corn starch with phenolic acids has received comparatively limited attention.

Among phenolic acids present in whole grains, ferulic acid (FA) is the most abundant, and corn is particularly enriched in FA relative to other grains. It is the predominant hydroxycinnamic acid found in the cell walls of corn kernels. Structurally, its phenolic hydroxyl and carboxyl groups serve as reactive sites for esterification or etherification with polyhydroxy polymers. Ferulic acid (FA) can form stable covalent linkages with polysaccharide chains through esterification reactions [16], thereby enhancing the compactness and stability of the polymer network. In cereal grains, FA is commonly bound to cell-wall polysaccharides, such as arabinoxylans, via ester or ether bonds [17]. These interactions are mainly attributed to the affinity between the carboxyl groups of FA and the abundant hydroxyl groups present on polysaccharide chains. Therefore, high-amylose corn starch, which contains numerous hydroxyl groups, may similarly undergo covalent grafting with FA under suitable conditions. Covalent grafting of FA has also been widely reported in various biopolymer systems, including egg white protein [18], β-lactoglobulin [19], rice glutelin, soy protein isolate [20], rice bran protein hydrolysates [21], and flaxseed protein isolate [22]. However, studies on the covalent modification of high-amylose starch with FA remain limited, particularly regarding the effects of such modification on the molecular organization and digestive behavior of the starch matrix.

Based on these considerations, this study utilized HACS and FA as raw materials to synthesize HACS-FA covalent grafts via a controllable wet-heat treatment under high temperature and pressure. The resulting chemical structures were validated using FTIR, ^1^H NMR, and other advanced techniques. The impacts of FA grafting were evaluated through complementary analyses, including SEM, DSC, TGA, in vitro digestion assays, and molecular docking. Additionally, this research not only provides direct evidence for the covalent modification of starch by phenolic acids but also offers technological insights into reducing digestibility and enhancing the processing performance of HACS by modulating treatment conditions. These results provide mechanistic guidance for designing corn-starch ingredients with reduced digestibility and tunable structure.

**Table 1:** Representative studies on starch modification with phenolic acids/polyphenols.

Starch Source	Phenolic Acid/ Bioactive Compound	Modification Method	Main Findings	Reference
Potato starch and maize amylopectin	Phenolic acids	Complexation	Changed starch structure, rheological properties, and digestibility	Li et al., 2018 [23]
Rice starch	Gallic acid	Non-covalent complexation	Increased resistant starch and decreased predicted glycemic index	Chi et al., 2019 [24]
Pea starch	Gallic acid	High-pressure homogenization-assisted complexation	Promoted starch-gallic acid complexation, delayed starch digestion	Villanova et al., 2022 [25]
Starch slurry	Caffeic acid	Heating-cooling inclusion complexation	Formed starch–caffeic acid inclusion complexes, slowed enzymatic digestion	Chi et al., 2022 [26]
Cassava starch	Ferulic, p-coumaric, sinapic acids	Esterification	Reduced digestibility and predicted glycemic index	Xu et al., 2022 [27]
Porous starch	Ferulic acid	Esterification	Improved antioxidant capacity	Zhang et al., 2022 [28]
High-amylose corn starch	Curcumin/Capsaicin	V-amylose inclusion complexation	Entrapped phenolic bioactives, reduced intestinal starch breakdown	Tarazi Riess et al., 2022 [29]
Corn starch	Gallic acid/Quercetin	Free radical grafting	Enhanced resistance to enzyme digestion; altered pasting properties	Wu et al., 2022 [30]
Rice starch	Quercetin/Other polyphenols	Inclusion complexation	Enhanced resistance to digestion, especially quercetin	Deng et al., 2023 [31]

## 2. Materials and Methods

### 2.1. Materials

Ferulic acid (FA; purity ≥ 98%) and high-amylose corn starch (HACS; purity ≥ 98%; amylose 72%) were obtained from Shanghai Yuanye Biotechnology Co., Ltd. (Shanghai, China) and Shanghai Macklin Biochemical Technology Co., Ltd. (Shanghai, China), respectively. α-Amylase, pepsin, and pancreatin were sourced from Sigma-Aldrich (Shanghai, China). Unless otherwise stated, all reagents were of analytical grade, and distilled water was used throughout.

### 2.2. Preparation of HACS-FA

HACS and FA were mixed in a 1:1 molar ratio (based on glucosyl units), and distilled water was added. The mixture underwent hydrothermal treatment at 111 °C and 0.15 MPa for 60 min. After the reaction, the mixture was cooled to room temperature, dried at 40 °C to constant weight, ground, and sieved through a 100-mesh filter. The product was washed three times with 95% ethanol (4 parts ethanol to 1 part sample) to remove unreacted FA, then freeze-dried (Alpha 2-4 LSC plus; Martin Christ, Osterode am Harz, Germany), and sieved again. The final product was stored at −20 °C and designated as HACS-FA. The grafting degree of FA onto HACS under these conditions was experimentally determined to be 53.1%. In parallel, a gelatinized starch control (HACS-P) was prepared by subjecting HACS to identical hydrothermal conditions in the absence of FA. In addition, a physical mixture of HACS and FA was prepared at a 1:1 molar ratio (based on glucosyl units) without covalent reaction and is hereafter referred to as HACS/FA-M.

### 2.3. Fourier Transform Infrared Spectroscopy (FTIR)

FTIR spectra were acquired on an FTIR spectrometer (Nicolet iS 50, Thermo Fisher Scientific, Waltham, MA, USA) according to Liu et al. [32]. For sample preparation, 1–2 mg of powder was homogenized with spectroscopic-grade anhydrous KBr (Aladdin Biochemical Technology Co., Ltd., Shanghai, China) and compressed into transparent pellets using a hydraulic tablet press (YP-15, Zhongshi Walk (Tianjin, China) Technology Development Co., Ltd., Wuhan, China) at 5 MPa for 120 s. Spectra were collected over the range of 400–4000 cm^−1^ with a resolution of 4 cm^−1^, and each sample was scanned 64 times.

### 2.4. ^1^H Nuclear Magnetic Resonance Spectroscopy (^1^H NMR)

^1^H NMR spectra of the samples were acquired using an NMR spectrometer (AVANCE III HD 500 MHz, Bruker, Fällanden, Switzerland) according to the method of Kenar et al. [33] with minor modifications. Briefly, 2.5 mg of the sample was dissolved in 0.7 mL of deuterated dimethyl sulfoxide (DMSO-d6) containing 0.1% (*v*/*v*) deuterated trifluoroacetic acid (CF3COOD). The mixture was stirred at 80 °C until a clear solution was formed. Spectra were acquired at 25 °C, and chemical shifts (δ) were referenced to tetramethylsilane (TMS) and reported in ppm.

### 2.5. Visible Absorption Spectroscopy

Visible absorption spectra were collected using a UV-Vis spectrophotometer (UV-2600, Shimadzu, Kyoto, Japan) following Huang et al. [34]. Briefly, 1 mg of the sample was dispersed in anhydrous ethanol (20 mL; 50 μg/mL), and iodine reagent was added to develop color prior to measurement. Absorbance was recorded over the range of 575–775 nm, which corresponds to the characteristic visible absorption of starch–iodine complexes.

### 2.6. Fluorescence Spectroscopy

Fluorescence measurements were conducted on a fluorescence spectrophotometer (RF-6000, Shimadzu, Kyoto, Japan) according to Chi et al. [24]. Briefly, 1 mg of the sample were prepared in distilled water to obtain a final concentration of 0.5 mg/mL. Spectra were collected at an excitation wavelength of 370 nm with a 5 nm slit width, and emission profiles were recorded.

### 2.7. Scanning Electron Microscopy (SEM)

The microstructure of HACS, HACS-P, and HACS-FA was examined by field-emission scanning electron microscopy (SU8010, Hitachi, Tokyo, Japan) in accordance with Pang et al. [35]. Samples were affixed to metal stubs with conductive carbon tape and gold-sputtered to improve surface conductivity. Imaging was performed at an accelerating voltage of 5–10 kV.

### 2.8. Differential Scanning Calorimetry (DSC)

Differential scanning calorimetry was performed on a DSC 250 system (TA Instruments, New Castle, DE, USA) according to Zheng et al. to characterize the thermal properties of HACS, HACS-P, and HACS-FA [36]. Briefly, 6 mg of the sample was accurately weighed into a 40 μL aluminum pan, and 20 μL of deionized water was added. The samples were sealed in pans using Tzero lids and left to equilibrate at ambient temperature overnight. Thermal scans were then run from 30 to 130 °C at 5 °C/min under a nitrogen flow of 20 mL/min, with an empty aluminum pan serving as the reference.

### 2.9. Thermogravimetric Analysis (TGA)

Thermogravimetric analysis was carried out on a TGA 8000 instrument (PerkinElmer, Waltham, MA, USA) according to Raza et al. to assess the thermal stability of HACS, HACS-P, and HACS-FA [37]. Approximately 10 mg of sample was weighed into an alumina crucible and heated from 30 to 600 °C at 10 °C/min under a nitrogen atmosphere. Record weight loss data throughout the heating process.

### 2.10. In Vitro Digestion

A standardized INFOGEST 2.0 static model was applied to mimic oral, gastric, and small-intestinal digestion of HACS, HACS-P, and HACS-FA [38]. In the oral stage, 5 g of sample was mixed with simulated salivary fluid containing CaCl_2_ and α-amylase (75 U/mL). The pH was adjusted to 7.0, and the mixture was incubated at 37 °C with shaking for 2 min. For the gastric phase, simulated gastric fluid containing pepsin was introduced, the pH was set to 2.0, and digestion was carried out at 37 °C for 120 min. Subsequently, simulated intestinal fluid supplemented with pancreatin (100 U/mL) and bile salts was introduced. The pH was adjusted to 7.0, and the intestinal digestion phase was carried out at 37 °C for 300 min. Aliquots were taken at intervals of 0, 10, 20, 30, 60, 90, 120, 180, and 240 min. Enzyme activity was halted by adding anhydrous ethanol. After centrifugation (4000 rpm for 10 min), glucose levels in the supernatant were measured using the glucose oxidase assay [39]. The hydrolysis percentage and the fractions of RDS, slowly digestible starch (SDS), and resistant starch (RS) were calculated using the equations below.

(1) $$Hydrolysis\;rate\left(\%\right)=\frac{G_{t}\times 0.9}{G}\times 100$$

(2) $$RDS\left(\%\right)=\frac{(G_{20}-G_{0})\times 0.9}{TG}\times 100$$

(3) $$SDS\left(\%\right)=\frac{G_{120}-G_{20}}{TG}\times 100$$

(4) $$RS\left(\%\right)=\left(TG-RDS-SDS\right)\times 100$$

In these equations, G_t_ is defined as the glucose amount (mg) quantified at time t (min) during digestion. The factor 0.9 is used for stoichiometric conversion from glucose to starch equivalents. G (mg) denotes the initial starch mass. G_0_, G_20_, and G_120_ denote the glucose masses (mg) determined at 0, 20, and 120 min, respectively. TG refers to the total starch amount (mg) present in the digestion system.

### 2.11. Molecular Docking

Docking calculations were performed in AutoDock Vina (v1.2.0) to examine the FA and α-amylase interaction. FA was prepared from the PubChem entry CID 445858, and the α-amylase receptor was sourced from the RCSB PDB (1PIF). Prior to docking, the protein and ligand were prepared using AutoDockTools (v.1.5.7) by adding non-polar hydrogen atoms and Gasteiger charges. The docking active site was centered at coordinates x = −0.15 Å, y = 2.25 Å, and z = 2.55 Å, with a grid box size of 75 × 90 × 65 Å. A semi-flexible docking protocol was employed, and the binding configurations were visualized and analyzed using PyMOL (v.2.5; Schrödinger, New York, NY, USA) and LigPlot+ (v.2.2).

### 2.12. Statistical Analysis

All measurements were performed in triplicate, and data are presented as the mean ± standard deviation. Statistical analyses were carried out using SPSS Statistics 26.0 (IBM, New York, NY, USA). The statistical significance of differences between means was assessed at *p* < 0.05. Figures were generated using Origin 2021 (OriginLab, Northampton, MA, USA).

## 3. Results and Discussion

### 3.1. FTIR

**Figure 1:**
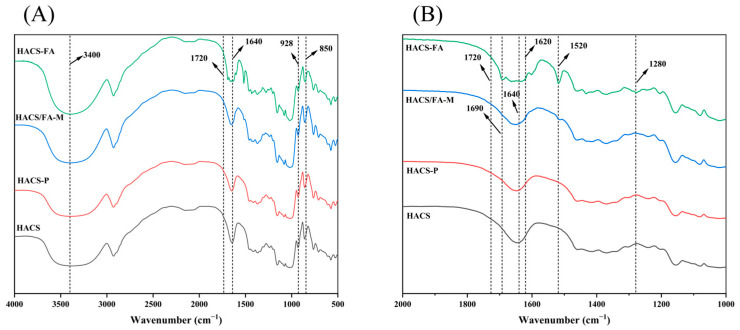
FTIR spectra of different samples. (**A**) FTIR spectra of high-amylose corn starch (HACS), ferulic acid-grafted high-amylose corn starch (HACS-FA), gelatinized starch control (HACS-P), and the physical mixture of HACS and ferulic acid (HACS/FA-M); (**B**) enlarged view of the selected spectral region.

As illustrated in the overall and enlarged views in Figure 1, there were no significant changes in the infrared spectra of HACS compared to HACS-P, indicating that the heat treatment process did not introduce new functional groups into the HACS backbone. By comparing the spectra of HACS and HACS-FA, changes in the intensity and shape of the broad absorption band around 3400 cm^−1^ were observed after FA grafting. These changes may be associated with alterations in hydrogen-bonding interactions, the introduction of hydroxyl-containing FA molecules, and the formation of covalent ester linkages between HACS and FA. Compared with HACS, HACS-P, and HACS/FA-M, HACS-FA showed a weak band near 1690 cm^−1^, which can be related to C=O stretching of FA-derived carbonyl groups. The band around 1520 cm^−1^ was attributed to the aromatic ring vibration of FA, while the absorption near 1640 cm^−1^ may be associated with bound water and C=O-related vibration. Slight spectral differences in the 1200–1100 cm^−1^ region were also observed, corresponding to C–O stretching vibrations [40].Together with the changes in the O–H stretching region discussed above, these spectral features indicate changes in the functional groups of HACS after FA modification. Similar to the reports of Fang et al. [41] and Mu et al. [15], simple physical mixing or non-covalent interactions between starch and phenolic compounds generally do not produce new characteristic FTIR bands. Therefore, the differences observed between HACS-FA and HACS/FA-M suggest that wet-heat treatment changed the chemical environment of HACS-FA rather than only producing a physical mixture. Furthermore, the appearance of FA-related bands in HACS-FA, especially those associated with carbonyl and aromatic ring vibrations, indicates that FA-derived groups were introduced into the starch system after wet-heat treatment. Together with the differences between HACS-FA and HACS/FA-M, these results suggest that FA modification changed the chemical environment of HACS, providing supporting evidence for the formation of HACS-FA.

### 3.2. ^1^H NMR

**Figure 2:**
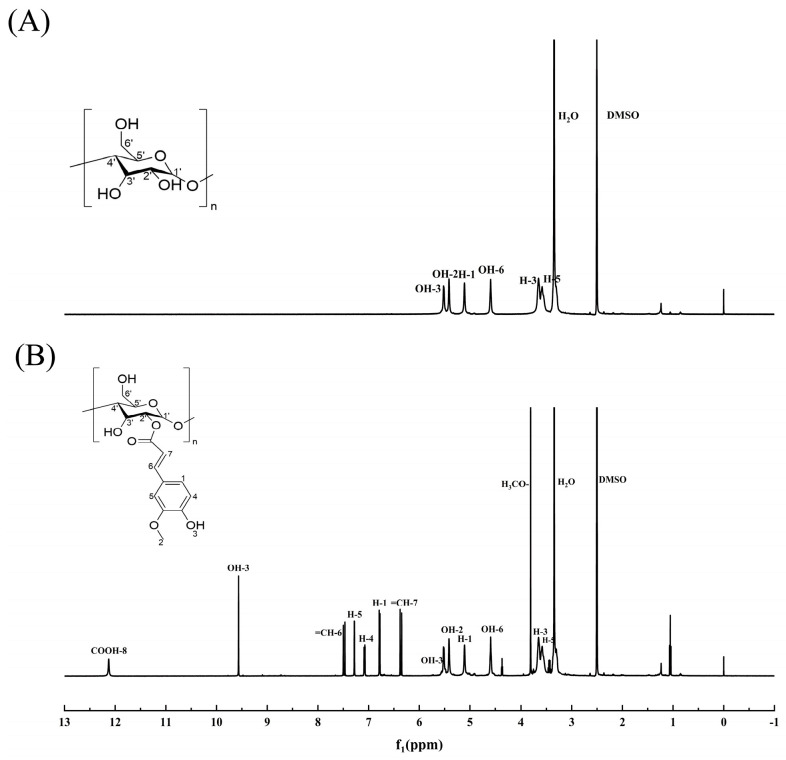
^1^H NMR spectroscopy of HACS (**A**) and HACS-FA covalent (**B**).

^1^H NMR spectroscopy was used to investigate the chemical structure of HACS-FA and provide information on possible interaction sites between HACS and FA. The ^1^H NMR spectrum of HACS (Figure 2A) displays characteristic glucosyl proton signals at 3.66 ppm (H-3) and 3.58 ppm (H-5). The OH-6 resonance is detected at 4.57 ppm, and the anomeric H-1 signal occurs at 5.11 ppm. In addition, two hydroxyl resonances at 5.50 and 5.40 ppm are assigned to OH-3 and OH-2 on the starch ring, respectively [42]. The 1H NMR spectrum of HACS-FA (Figure 2B) retains the characteristic signals of the HACS backbone in the 3.0–5.6 ppm range with largely unchanged chemical shifts, suggesting that the core structure of HACS remained intact during modification. Upon covalent binding of FA, several changes were observed in the HACS-FA spectrum. Compared to native HACS, the peak at 3.81 ppm in HACS-FA increased, which was attributed to the methoxy group protons from the FA moiety. Notably, the signal intensity assigned to OH-2 at 5.40 ppm decreased in HACS-FA, suggesting that hydroxyl groups in this region may be involved in esterification and that the amount of free hydroxyl protons was reduced. This interpretation aligns with Yong et al. [43], where attenuation of selected functional-group signals was taken as evidence of their participation in conjugation. Moreover, the ^1^H NMR spectrum of HACS-FA exhibited the characteristic signals of the FA moiety, including the alkene protons (at 7.68 ppm and 6.38 ppm), the phenyl ring structure (at 6.79 ppm, 7.09 ppm, and 7.28 ppm), and the methoxy group (at 3.81 ppm). Interestingly, the characteristic carboxyl group signal of FA at 12.14 ppm was weakened in the HACS-FA spectrum. While previous studies, such as Liu et al. [44], reported the complete disappearance of phenolic acid carboxyl signals upon conjugate formation, a faint carboxyl signal remained detectable in this study. This may be explained by the heat treatment process during the reaction, which disrupted the original starch granule structure and created surface indentations or micro-holes. Consequently, a minor amount of FA may have been physically entrapped within these starch cavities without undergoing a chemical reaction, leading to the observed residual carboxyl signal. In summary, the weakening of the OH-2-related signal of HACS and the concomitant presence of FA-derived aromatic and alkene signals support the formation of ester linkages between FA and HACS. These findings provide supporting evidence for the covalent grafting of FA onto HACS and are consistent with the FTIR results.

### 3.3. Visible Absorption and Fluorescence Spectroscopy

**Figure 3:**
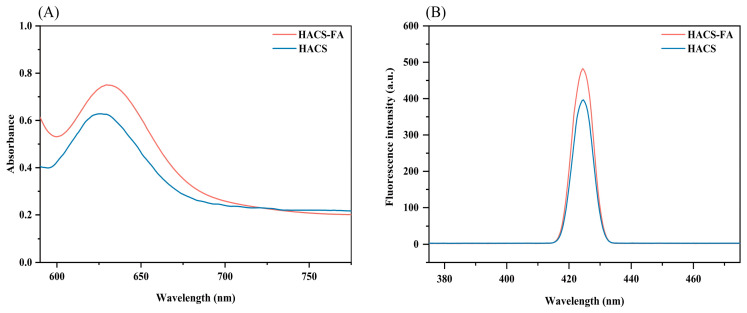
Visible absorption and fluorescence spectra of different samples. (**A**) Visible absorption spectrum (**B**) Fluorescence Spectrum.

As illustrated in Figure 3A, the visible absorption spectra of HACS and HACS-FA exhibited similar profiles, with both showing a maximum absorption wavelength (λmax) around 625 nm, characteristic of starch-iodine complexes. This similarity suggests that the fundamental structural features of HACS are largely retained in the HACS-FA conjugate, in good agreement with the FTIR and NMR results described above. However, the absorption spectrum of HACS-FA shows an overall bathochromic shift compared to native HACS. This shift is likely due to the covalent grafting of FA onto HACS chains, which extends the conjugated system and lowers the energy required for π–π* transitions. The visible absorption of starch-iodine complexes is closely related to the helical structure and iodine-binding ability of starch chains. Previous studies reported that amylose-iodine complexes show strong absorption around 600–620 nm, and the maximum absorption wavelength can vary with starch chain length and molecular organization [45,46]. In the present study, HACS and HACS-FA both showed a maximum absorption wavelength around 625 nm, indicating the formation of starch-iodine complexes. Compared with HACS, the maximum absorption wavelength of HACS-FA shifted slightly to a longer wavelength, suggesting that FA modification affected the interaction between starch chains and iodine. This change may be related to alterations in chain arrangement or the local molecular environment of HACS after FA modification.

Fluorescence spectroscopy was further used as a complementary method to examine the emission response of HACS and HACS-FA under identical measurement conditions [47]. As shown in Figure 3B, both HACS and HACS-FA exhibited an emission maximum in the range of 420–430 nm upon excitation at 370 nm. Since native starch generally has weak intrinsic fluorescence, the fluorescence data in this study were mainly used to compare the relative emission difference between HACS and HACS-FA, rather than to directly confirm covalent grafting. Previous studies have shown that fluorescence spectroscopy can be used to evaluate the response of starch-small molecule complexes and to reflect changes in the local microenvironment of starch-based systems [48,49]. Compared with native HACS, HACS-FA showed higher fluorescence intensity, which may be related to the introduction of FA and the formation of ester linkages. The aromatic structure of FA and the altered local electronic environment of the starch chains after grafting may contribute to the enhanced emission response. Thus, the increased fluorescence intensity provides supporting evidence for changes in the electronic microenvironment of HACS after FA grafting, which is consistent with the FTIR and ^1^H NMR results.

### 3.4. SEM

**Figure 4:**
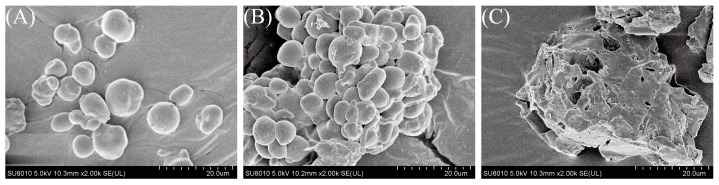
SEM images of high-amylose corn starch (HACS) (**A**), gelatinized starch control (HACS-P) (**B**), and ferulic acid-grafted high-amylose corn starch (HACS-FA) (**C**).

Figure 4A shows that HACS exhibits a typical polygonal or irregular spherical structure, with smooth surfaces and intact contours, showing no obvious damage or holes, which reflects its naturally dense granule morphology. After gelatinization treatment, as seen in Figure 4B, the starch granule interfaces become blurred, and the granules aggregate. This indicates disruption of the crystalline regions and double helix structure, with full swelling and fusion of the molecular chains. In contrast, HACS-FA (Figure 4C) presents a significantly different microscopic morphology. The sample surface is rough, with noticeable grooves and cross-linked textures, and local pores are enlarged, with multilayer stacking and amorphous aggregation regions visible. Surface pore enlargement is probably driven by water uptake and swelling during heating, which may allow FA to diffuse more readily into the granule interior [12]. These results indicate that FA incorporation during grafting reorganized starch chain packing, leading to a loss of the original structural order. Similar observations were reported by Chen et al., who found that tannic acid increased microstructural disorder in starch, consistent with our results [50].

### 3.5. Thermodynamic Properties

Thermal transitions of HACS, HACS-P, and HACS-FA were characterized by DSC, and the corresponding parameters are presented in Table 2. Native HACS exhibited a gelatinization peak temperature (Tp) of 95.42 °C, while the onset (To) and conclusion (Tc) temperatures were 63.59 °C and 102.51 °C, respectively. The relatively high Tp value is consistent with the thermal behavior of high-amylose maize starch, whose ordered crystalline regions and compact chain packing generally require higher energy for gelatinization than normal maize starch [51,52]. The gelatinization enthalpy (ΔH) was 10.38 J/g, suggesting that the native starch granules had a well-organized crystalline structure. After gelatinization, the thermal transition temperatures of HACS-P remained largely unchanged. However, ΔH decreased to 9.51 J/g, suggesting that heat treatment partially disturbed the crystalline domains of starch. For HACS-FA, covalent grafting exerted a significant impact on its thermal properties, with Tp decreasing to 90.20 °C and ΔH declining to 8.34 J/g (*p* < 0.05). The onset temperature (To) of HACS-FA was slightly higher than that of native HACS, likely due to restricted starch-chain mobility and altered starch-water interactions after FA grafting, which may delay the initial swelling of less stable crystalline regions. Similar effects have been reported in starch-polyphenol and other starch-component systems [53,54]. The subsequent decreases in Tp and ΔH suggest that FA grafting also partially disrupted the crystalline order, reducing the overall energy required for gelatinization. Jiang et al. [55] observed similar thermodynamic changes when chlorogenic acid was complexed with lotus seed starch via microwave heating, showing that these treatments disrupt the crystalline structure and decrease thermal stability. Accordingly, we propose that FA grafting perturbs crystalline order in HACS granules, thereby facilitating gelatinization and lowering thermal stability relative to native HACS. Furthermore, Chen et al. [56] found that starch-polyphenol complexes weaken the crystalline structure of starch granules. This decreases the energy needed to disrupt double helices, shifts gelatinization to lower temperatures, and promotes gelatinization. This trend is consistent with the present findings. Previous work has also indicated that coupled interactions among polyphenols, water, and starch molecules may promote gelatinization [57]. Taken together, these results confirm that the covalent integration of FA modified the internal molecular structure and thermodynamic properties of HACS.

**Figure 5:**
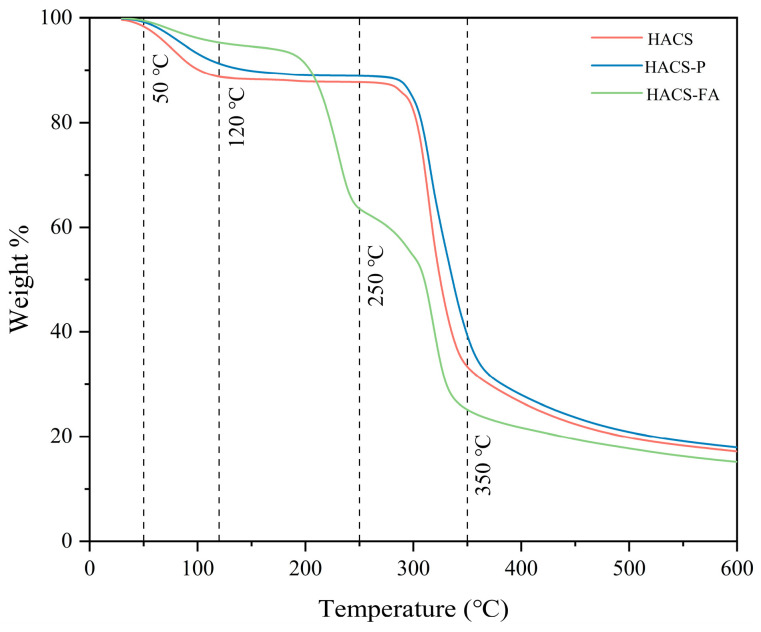
Thermogravimetric Analysis Curves of HACS, HACS-P, and HACS-FA.

TGA provides a practical means to assess starch thermal stability and its interactions with coexisting components [27]. As illustrated in Figure 5, HACS shows the highest mass-loss rate between 50 and 120 °C. This suggests that native granules mainly contain free or weakly bound water, which is readily removed upon heating. In contrast, HACS-P and HACS-FA adsorb more bound water, with a higher temperature required for dehydration, resulting in a slower initial weight loss. After entering the main thermal decomposition stage (approximately 250–350 °C), HACS shows higher decomposition temperatures and a slower weight loss rate, while HACS-FA undergoes noticeable thermal degradation earlier. Phenolic compounds have been shown to bind to adjacent starch chains, weakening the interaction between them and subsequently reducing their thermal stability [58]. Chen et al. further reported that several phenolic compounds (e.g., protocatechuic acid, ellagic acid, naringin, and tannic acid) markedly influence the thermal stability of corn starch [50]. Therefore, it can be inferred that the covalent binding of HACS and FA weakens the intermolecular forces and ordered structure, thereby reducing overall thermal stability. Mao et al. reported a comparable trend, where phenolic acid addition decreased the thermal stability of potato starch [59]. Meanwhile, this trend is consistent with the DSC results showing reduced thermal stability of HACS-FA. Overall, grafting with ferulic acid induced structural modifications, lowered the energy required for gelatinization, and led to earlier thermal decomposition, which agrees with the SEM observations described above.

### 3.6. Digestive Properties

**Figure 6:**
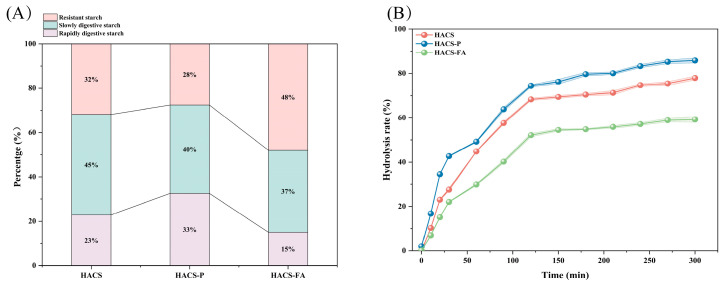
In vitro simulated digestive percentage of different types of starch after hydrolysis (**A**) and hydrolysis rate (**B**) for different time periods of HACS, HACS-P, and HACS-FA.

The digestibility of HACS, HACS-P, and HACS-FA was evaluated in a simulated gastrointestinal model incorporating salivary, gastric, and intestinal fluids. As shown in Figure 6A,B, the starch-fraction profiles and hydrolysis curves differ across treatments, with statistically detectable effects on digestion behavior (*p* < 0.05). Meanwhile, the proportion of RDS also exceeded 33%, further confirming its characteristic of being more easily rapidly digestible. Heat treatment likely enhances granule hydration and swelling, thereby exposing more substrate surface for digestive enzymes [60,61]. In native HACS, only 23% of starch was classified as rapidly digestible starch, consistent with slower hydrolysis. By contrast, gelatinization markedly increased the digestion rate. HACS-FA exhibited a markedly different digestion profile from HACS-P, with RDS reduced to 15% and resistant starch (RS) increased to 48% (*p* < 0.05). The change in digestibility of HACS-P was mainly due to the structural disruption caused by gelatinization, while under the same gelatinization conditions, the digestibility of HACS-FA decreased instead of increasing, indicating that covalent grafting of FA brought additional resistance to digestion. Raza et al. also made similar findings. They found that the digestion rate of rice starch-ferulic acid complexes, obtained after ultrasonic and high-pressure homogenization treatment, was significantly reduced. However, when chlorogenic acid was mixed with starch, it had no effect on the interaction between starch chains and digestive enzymes. The results suggest that the complexation between starch and ferulic acid may affect the action of digestive enzymes on starch [62]. In addition, Zheng et al. reported that the reduction in digestion rate and RDS content in FA-corn starch complexes, formed by heat treatment and extrusion processing, was mainly due to FA’s inhibition of amylase activity during starch hydrolysis, which suppressed the rapid increase in digestibility [63]. The digestion profile of HACS-FA is attributable to interactions between FA and starch chains, particularly esterification. This modification may reduce enzyme accessibility to hydrolysis-prone sites and thereby increase resistance to enzymatic degradation [14]. Previous studies have demonstrated that polyphenols (such as *Glochidion wallichianum* extract, coumaric acid, caffeic acid, catechins, and proanthocyanidins) can inhibit the formation of crystalline structures by interacting with starch molecules (such as brown rice starch, corn starch, and chestnut starch) through hydrogen bonds and van der Waals forces, thereby promoting short-range ordered structures. This, in turn, synergistically regulates the retrogradation properties and digestibility of starch [64,65,66]. These structural modifications and the resulting increase in resistant starch content are of potential nutritional and health significance, as higher RS intake has been linked to improved postprandial glucose regulation, enhanced colonic fermentation, and beneficial effects on gut microbiota composition. Therefore, HACS-FA may be a promising functional ingredient for foods designed to help moderate postprandial glycemic response and support intestinal health.

### 3.7. Molecular Docking

Simulated in vitro digestion showed that covalent grafting of HACS with FA markedly reduced the digestion rate of the modified starch. Free FA has been reported to inhibit α-amylase activity involved in starch digestion [67]. However, for HACS-FA, the reduction in digestibility may reflect both structural changes in the starch matrix after covalent grafting and the possible enzyme-related effects of FA, while their relative contributions cannot be fully distinguished in the present study. To probe the possible enzyme-related mechanism, molecular docking was performed to model the interaction between free FA and α-amylase.

**Figure 7:**
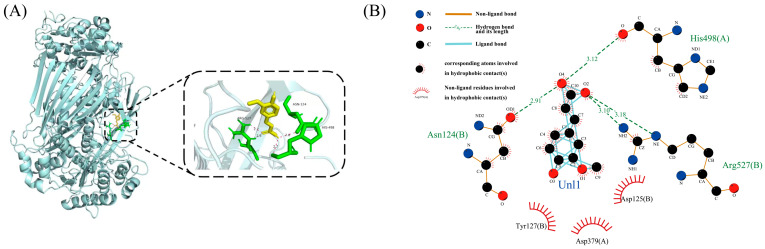
Molecular docking results of FA and α-amylase. (**A**) Schematic representation of the binding mode of FA with α-amylase; (**B**) Docking analysis results showing the interaction details.

Docking (Figure 7) suggests that free FA associates with α-amylase via hydrogen bonding and hydrophobic contacts. FA was predicted to be located near the substrate-binding region of α-amylase, where it may affect catalytic accessibility and starch binding [36]. Hydrogen bonds were predicted between the phenolic hydroxyl group of FA and residues including Asn124, His498, and Arg527. In addition, FA is stabilized by van der Waals interactions and π–π stacking interactions with nearby residues, including Tyr127 and Asp125 [68]. This is generally consistent with previous studies showing that phenolic acids may inhibit α-amylase by interacting with residues located in or near the enzyme active site. For example, Shi et al. [69] found that hydroxybenzoic acid (PHBA) and FA inhibit α-amylase by occupying the active site.

Nevertheless, the docking results describe the potential interaction between free FA and α-amylase and should not be directly interpreted as the dominant mechanism for the reduced digestibility of HACS-FA. In HACS-FA, FA is covalently linked to the starch matrix through ester bond formation, as supported by the FTIR and ^1^H NMR results. This grafting may alter the molecular organization of HACS and reduce the accessibility of hydrolysis-prone sites to digestive enzymes [70]. Therefore, compared with free FA, grafted FA is expected to have fewer opportunities to directly interact with α-amylase. Notably, after covalent grafting, FA, being embedded in the matrix of high-amylose starch, has significantly reduced opportunities to interact with the enzymes, thus no longer being the primary factor determining digestibility. Overall, the reduced digestibility of HACS-FA is more likely associated with structural remodeling caused by covalent grafting, while a contribution from enzyme-related interactions of FA cannot be completely excluded.

## 4. Conclusions

This study successfully constructed a covalently grafted HACS-FA structure under controlled moist heat treatment conditions (111 °C, 0.15 MPa, 60 min), and systematically investigated its structural changes and their impact on digestion behavior. Esterification between HACS and FA embeds FA into the dense crystalline region of HACS, causing starch granules to transform from their original elliptical structure to a rough, porous, and lamellar amorphous morphology, significantly reducing the gelatinization enthalpy and weakening thermal stability. Correspondingly, the RDS component of HACS-FA is significantly reduced, the RS content increases, and the overall digestibility further decreases. Docking suggests that free FA can interact with α-amylase through hydrogen bonding and hydrophobic interactions. By contrast, once FA is covalently grafted, it becomes embedded within the HACS matrix, which limits its exposure to the enzyme and weakens the interaction.

It is noteworthy that although daily cooking of corn may trigger a partial reaction between starch and ferulic acid, the difficulty in precisely controlling temperature, moisture, and time during cooking makes quantitative analysis of the resulting structure difficult, and the occurrence and extent of cross-linking are hard to determine. In contrast, the controlled moist-heat strategy employed in this study stably induced the covalent grafting reaction under controlled conditions, providing reliable experimental evidence for elucidating the relationship between structural changes, thermal properties, and digestive behavior. From an application perspective, HACS-FA may affect product quality because FA grafting changed the morphology and thermal behavior of HACS. These changes may influence water absorption, paste stability, baking performance, freeze/thaw stability, syneresis, texture, and indirect sensory properties in real food systems. Therefore, when HACS-FA is used as a functional ingredient, the addition level, water ratio, heating conditions, and formulation compatibility with proteins, lipids, or hydrocolloids should be optimized to balance reduced digestibility with acceptable product quality. This study is also limited by the use of a static in vitro digestion model, which does not fully represent real gastrointestinal conditions and does not include colonic fermentation or gut microbiota effects. Future studies should therefore evaluate HACS-FA in real food matrices, dynamic digestion and fermentation models, and further examine its clean-label suitability, FA stability during processing and storage, and possible in vivo oxidative products such as quinone-type or smaller phenolic degradation products. In summary, this study demonstrates the feasibility of covalently grafting phenolic acids onto HACS and provides guidance for developing corn-based ingredients with low digestibility and tunable structure.

## Figures and Tables

**Table 2:** Thermal properties of high-amylose corn starch (HACS), gelatinized starch control (HACS-P), and ferulic acid-grafted high-amylose corn starch (HACS-FA).

Samples	To ^1^ (°C)	Tp ^1^ (°C)	Tc ^1^ (°C)	ΔH ^1^ (J/g)
HACS	63.59 ± 1.73 ^b^	95.42 ± 2.27 ^a^	102.51 ± 2.48 ^a^	10.38 ± 0.90 ^a^
HACS-P	64.34 ± 1.68 ^b^	94.04 ± 1.98 ^a^	101.77 ± 2.43 ^a^	9.51 ± 0.68 ^a^
HACS-FA	67.24 ± 1.20 ^a^	90.20 ± 0.64 ^b^	96.97 ± 0.20 ^b^	8.34 ± 0.47 ^b^

^1^ To, onset temperature; Tp, peak temperature; Tc, conclusion temperature; ΔH, gelatinization enthalpy. Results are expressed as mean ± standard deviation. Different letters within the same column indicate significant differences (*p* < 0.05).

## Data Availability

The original contributions presented in the study are included in the article; further inquiries can be directed to the corresponding authors.
